# Leader–member exchanges with leaders who have worked for 25 years in health institutions

**DOI:** 10.1002/nop2.1616

**Published:** 2023-01-29

**Authors:** Frøydis Vasset, Lisbeth Fagerstrøm, Marianne Frilund

**Affiliations:** ^1^ Department for Health University college in Moldes Molde Norway; ^2^ Faculty of Education and Welfare Studies Åbo Akademi University Turku Finland; ^3^ Department of Health Sciences Ålesund Norwegian University of Science and Technology Norway

**Keywords:** hospitals, leader, manager, municipality, nurse, relationships

## Abstract

**Aim:**

The aim is to investigate long‐term leader experiences with leader– member exchanges (LMX) over 25 years. Leader–member exchanges focus on relational power and communication exchanges between leaders and employees when they communicate with each other or perform an action.

**Design:**

This qualitative study is characterized by a phenomenological hermeneutical design and is based on the informants' interpretation and construction of meaning.

**Method:**

A qualitative study with eight interviews with supreme nurse leaders from the Norwegian and Finnish health care services.

**Result:**

The data analysis and interpretation show that relationships are built through trust, dialogue and confirmation and are affected by other contextual aspects such as the organizational size or workload and human factors such as safety, angst, and self‐esteem. The informants fight for their subordinates. Interprofessional management and obtaining good relations with the doctors was challenging. No Patient or Public Contribution

## 1 INTRODUCTION

Nurse leaders in health institutions are responsible for approximately 50–70 employees. The Nordic countries' requirements for formal leadership training vary (Hafsteinsdóttir, 2019). In Norway, many leaders in municipal health services have only a bachelor's degree in clinical nursing, without a higher degree in leadership, while Finland requires leadership training at a master's or doctoral level (Hafsteinsdóttir, 2019; Vasset et al., 2012). Leaders in health organisations are widely believed to have strong potential in improving employees' and organisations' functions. Good leadership provides information that can be used in decision‐making and for employee and organisation training and development needs (Yukl, 2012). Nursing scientists state that nurses need to take leadership responsibility at all levels, be proactive, develop technological innovations and the tools to improve care and ensure that they are involved and participate in patients' and clients' interprofessional help (Lomborg, 2019). Salmela (2012) states that nurse leaders' primary responsibilities include leading staff, processes, and culture. Nursing leaders' goals are to create and achieve long‐term strategic visions to enable employees to change with the social context. The role of nursing leaders is to help employees develop a shared sense of mission and to inspire and motivate others (Frilund, 2015; Hafsteinsdóttir, 2019; Quinn, 2020; Salmela, 2019). To achieve these goals, leaders have high‐quality relations and the leader–member exchanges (LMXs) with the employees (Vasset et al., 2012). It will be easier for leaders to give these employees tasks that require trust between leader and subordinates. These subordinates can gain access to courses and further education. Research shows that leaders cannot have an equally good relationship with all subordinates. It is not everyone they see every day either. Low quality of relationships, and exchanges between parties may lead to poor communications (Vasset et al., 2012). That can be lacking information, not being invited to an important meeting, or receiving inaccurate and too little information.

## 2 BACKGROUND

LMX theory focuses on relational power and communication exchanges between leaders and employees when they communicate with each other or perform an action. It also focuses on employees' and leaders' attitudes and behaviours (Day & Miscenko, 2016; Vasset et al., 2012; Yukl, 2012). LMX theory has been tested empirically in numerous studies and refined over the years (Day & Miscenko, 2016; Vasset et al., 2012). LMX has its foundation in active factors for both leaders and subordinates—such as good contributions, mutual effects, and recognition—but also frustration, violation, and uncertain factors. All these factors correlate strongly with employee and leader job satisfaction (Bauer & Erdogan, 2016; Vasset et al., 2012). Researchers have found that leaders' and employees' views of their LMX relationships might differ (Bauer & Erdogan, 2016). This discrepancy also involves physical and mental efforts, emotional support, information, and encouragement from leaders. All parties in a working relationship contribute to developing and maintaining sociopsychological processes, such as self‐knowledge, interpersonal skills, and cultural competence (Martin et al., 2018; Vasset et al., 2012). LMX theory describes how Leader–member relationships may develop stepwise over time, starting with the initial interaction between the dyad members. This initial interaction is followed by a sequence of dyadic relationships and exchanges in conversations with subordinates in which individuals “test” one another to determine whether they can build relational expertise based on respect and high‐quality exchanges. If the exchange behaviour is positively received, the individuals continue with high‐quality LMXs; if not, the relationship is likely to remain at a lower LMX quality (Bauer & Erdogan, 2016; Davis & Gardner, 2004; Day & Miscenko, 2016; Vasset et al., 2012; Yukl, 2012). Theorists note that trust between the parties is essential if they are to converse with each other (Eraut, 2004). The outcome of LMX interactions depends on the relationship level (Vasset et al., 2012; Yukl, 2012). It may be necessary to introduce LMX theory in municipal health services and hospitals because LMX quality and work environment are correlated.

Health professionals who have high LMXs receive more attention, information, and resources from leaders and perform more complex and interesting tasks. They tend to reciprocate this favourable treatment with higher performance levels, loyalty, conversational honesty, and positive attitudes. LMX theory contains the main dimensions of communication, respect (complication), trust, and obligation (Bauer & Erdogan, 2016; Peterson & Aikens, 2017). High LMX in communication involves physical and mental effort, emotional support, useful and thorough feedback, and encouragement from leaders. All parties in the working relationship contribute to developing and maintaining sociopsychological processes, such as self‐knowledge, participation, confirmation, interpersonal skills, constructive discussion, and cultural competence (Bauer & Erdogan, 2016; Peterson & Aikens, 2017). One of the most important factors is being able to listen to others, especially those who need to be listened to (Lervik & Vasset, 2021).

In this context, the objective is to study the long‐term leader's experience with LMXs through 25 years as a leader at health institutions.

## 3 METHOD

### 3.1 Design

This qualitative study is characterised by a phenomenological hermeneutical design approach, accorded to Creswell (2014), Kvale et al., (2015). The study is based on the informants' interpretation and construction of meaning. Individual interviews were conducted with municipal health service and hospital leaders. The research adopted a retrospective approach to the experiences of long‐term leaders' views of LMX in their work (25 years). This study adopted an inductive approach and was conducted according to Creswell's (2014) method, which describes a holistic approach involving reflection and discovery.

### 3.2 Sample

The participants were supreme nurse leaders from the Norwegian and Finnish health care services, divided into two levels of health care, primary and specialist health services, and closely connected to each other.

The participants consisted of two nurse leaders from primary health services and two from specialist health services in Norway and Finland, resulting in eight participants. Table 1 shows the informants' formal educational level, additional education, generational affiliation, title, and nationality (Table 1). The concept of data saturation is firmly embedded within some qualitative research logic. Data saturation has also been identified as the most evoked justification for sample size in qualitative research in the health domain (Brown & Clarke, 2021; Vasileiou et al., 2018). Brown and Clarke (2021) are even cited as recommending that a minimum of 12 interviews is required “to reach data saturation, but according to themselves, they have not written that. We saw a saturation tendency already at eight interviews. It was an in‐depth interview, and the informants received the questions a week in advance.

**TABLE 1:** Study sample.

Informant	Formal education	Further education		Current title	Nationality
1N	Nurse	Leadership, master's degree	Specialist health service	Section leader	Norwegian
2N	Nurse	Leadership, economy	Primary health service	Section leader	Norwegian
3N	Nurse	Leadership, guidance pedagogy	Primary health service	Section leader	Norwegian
4N	Nurse	Leadership, master's degree	Specialist health service	Section leader	Norwegian
5F	Biomedical laboratory scientist.	Performance management, master's and PhD in health sciences	Specialist health service	Section leader	Finnish
6F	Nurse	Master's in management, lean education, expert nurse	Specialist health service	Section leader	Finnish
7F	Nurse	Expert nurse	Primary health service	Section leader	Finnish
8F	Nurse	Expert nurse, master's, and PhD in nursing sciences	Primary health service	Section leader	Finnish

Before the interview process started, specialist and primary health service leaders were contacted and informed about the study's purpose. We used the snowball‐sampling method to find the informants. We often received recommendations from someone in the health system. An interview guide was used to gather data about the nurse leaders' leadership styles and experiences over a long time. The first eight leaders we contacted were willing to participate in the study.

The inclusion criteria were as follows: the participants had to (1) have full‐time jobs, (2) be in their positions for approximately 25 years, and (3) speak Norwegian or Swedish. All participants were top leaders in primary or specialist health services in Norway or Finland. Participants were excluded if they had less than 25 years of management/leadership experience.

### 3.3 Data collection

The data were collected through individual interviews with an open interview guide. We conducted eight individual interviews with leaders in healthcare institutions. The exact number of interviews was conducted in Norway as in Finland. The interviews took approximately 1.5 h and were completed in the spring–summer 2020. In such narrative interviews, conversational depth was at the centre, and saturation was difficult to determine. The participants were initially asked to describe their LMX practices, education, and work history. They were subsequently asked about their perceptions of leadership styles and asked to talk about their leadership styles. Furthermore, they were asked to describe the factors influencing leadership. Each interview was approached individually, guided by the participants' responses.

### 3.4 Analysis

The analysis process was performed according to Malterud's (2017) textual condensation of four steps, which constituted the structure of our analyses:

1. The transcripts were read to obtain an overall impression of the respondents' experiences.
2. The transcripts were read in detail to identify meaningful themes.
3. The content of the themes was abstracted and coded to subthemes.
4. The essence of each code group was summarised and used to develop the basis of the Results section.

We found three main categories with underlining subcategories. Each main category had two subcategories.

### 3.5 Ethical considerations

Research Ethics Committee approval for this study was obtained from the Norwegian Social Science Data Services. Support was granted because no registry was created for the study, the analysis was anonymous, and it did not include any personal names. The participants were given written and verbal information about the purpose of the project. The informants were told that the interview was anonymised and would be deleted after the completion of the study. They could withdraw from the study at any time without giving a reason. Informed consent was obtained from the respondents according to the rules of the Helsinki Declaration. The exact number of interviews was conducted in Norway as in Finland. The interviews were open, so the whole story could emerge. A Dictaphone was used during the interviews.

## 4 RESULTS

The data analysis and interpretation results showed that relationships between people in an organisation were built through trust, dialogue, and recognition. All informants described challenges for the leaders, including their reality, by expanding upon their nursing intensity, work stress, new technology, economic realities, etc. From having been a leader with a personal relationship with their workers, the leaders moved far from the clinical work, and personal relationships with their employers were no longer possible.

### 4.1 Category 1: Relationship built through trust

This category has two subcategories: an internal relationship of trust between leaders and subordinates and an external relationship of trust caused by political decisions about organisational mergers and changes.

#### 4.1.1 Subcategory a: Trust and respect between leaders and subordinates

Trust and respect between leaders and subordinates are not self‐evident. The informants explained that trust and respect between people result from working or spending time together. The proposal of building trust must be a bilateral relational condition. The informants said that the Leader must trust the co‐workers and vice versa.

“As leaders, you cannot own each problem or each project; you must delegate responsibility and freedom to the employers” (*F6*). An essential component in building a trusting relationship is the employer's conviction that the Leader is everyone's Leader, independent of profession, merger, or new organisation. On days that processes were changed, the employer's conviction was more important than ever. “Do I want to be well taken care of by leader when I come from another department?” (*F5*). The quote shows the participant's wishes that needed to be met during the turbulence that prevails in health care.

Midcareer, and today, it is no longer possible “to do everything by yourself” (*F6*). Many events co‐occur, and the Leader must learn to trust her or his staff. To trusting someone and giving them responsibility takes time. It is not self‐evident that leaders create the opportunity of building a trusting relationship with their staff. The informants said they still wanted to be the ones who had everything in their hands and made decisions for their subordinates. The informants emphasised that building a trusting relationship was necessary to be a good leader and that it took time for them to realise this.

The informants also reflected on the advantage of not being from the municipality where they worked. “I was not friends with anyone I was to lead. And therefore no one is favoured” (*N3*). Some time was spent creating a common culture, especially in hospitals. “It was difficult for doctors to accept that nurses should lead them” (*N1*).

#### 4.1.2 Subcategory b: Trust in merged institutions

Because many organisations have merged, the staff has experienced increased insecurity. The new organisational structures can lead to bad relationships between the staff or prevent new relationships from being established. The informants described mistrust among some staff, especially if the Leader came from a different part of the organisation than where the subordinate worked. The informants described situations in which they questioned their place in the future organisation. The staff fear losing their jobs or being forced to change tasks and workplaces. If the employees also lacked insight into the significance of the change, the mistrust was even more significant.

“We spent a lot of time creating a common culture in the rehabilitation department” (*N1*). “The therapists thought I was just the leader of the nurses, so I had to work with this trust” (*N4*). The informants gave the impression that the hospital collaboration was not good. “We had conflicts with the therapists. Tension in the work” (*N4*).

Many processes run simultaneously and at different organisational levels. As a leader, it is a great honour to steer the business towards common goals, especially to create trusting relationships.

### 4.2 Category 2: Relationships are built through dialogue

Another critical factor in building relationships is the competence to create a dialogue between leaders and others in the organisation and to create a dialogue with politicians who have an extraordinary role in leading health care. A participant described her experiences contacting politicians. The result indicates two subcategories to this main category: dialogue is the art of listening to subordinates and choosing the right time and strategy to present their expectations to politicians.

#### 4.2.1 Subcategory a: Dialogue and the art of listening with subordinates

The informants said that good relationships are built through dialogue. The leaders should hold dialogues monthly with their employees. “Have you achieved the goal, what is the problem, what is the reason why the goal has not been reached?” (*F5*). Having good communication means everything in the departments. We listen to them, the informants said. “It was concerning, of course it was. I would be without work if not.” (*N1*). In the communications, the informants continually addressed the change intention, that is, what they intended to achieve. The informants said they had to return to where they were last time. The informants had a dialogue with their subordinates and discussed where they were in the process and what they must relate to today. “What is our vision for the work”? (*N4*). “What is the intention of the changes in the department, and what do we want to achieve with them”? The informants said they must help the staff see the whole healthcare picture, not simply their minds. However, how are all healthcare institutions affected? “The vision of the strategy means from our point of view” (*N4*). All the participants emphasised the importance of having functional relationships between individuals in and outside the organisations. As the organisations merged, the dynamics between people changed. Suddenly becoming part of a large organisation changed the relationship between the leaders and the employees.

The informants said that if the doctors were asked whether they worked in teams, “they say yes, yes, yes”. “You ask with whom; they say the pathologist, the radiologist, the internal medicine” (*N4*). The informants said that the doctors do not realise that they have other partners who are also professionals. The informants reflected on how they should interpret this. Furthermore, they thought they received criticism regarding how they should live with everything they heard. The informants thought they were good at ignoring what they heard in larger assemblies: “You can hear things about your yourself that are not true; I have become ‘thick‐skinned’” (*F2*).

The informants thought starting with the employees' performance appraisal was strange. “Some employees do not have goals and meaning associated with their job” (N1). “It is nice when someone comes to me and says, you are so good at wording yourself. I appreciate that” (*N1*).

Dialogue means more than informing. In dialogue, both partners are on the same level, and both are important. The art of listening is a fundamental skill that leaders need to develop. Without the ability to listen, no dialogue will result from only one‐sided communication resulting in frustration and work stress.

#### 4.2.2 Subcategory b: Dialogue with politicians

This subcategory creates prerequisites for “good care” to achieve common intentions and visions.

The informants said that in their leadership role, they also had contact and dialogue with politicians. “It is certainly different between countries, but at least in our place, we have been able to discourse with the political leadership” (*F6*). The leaders said they must listen to and read politicians' discourse carefully, understand what they mean, and see what is possible. Then, they must learn to take advantage of opportunities when they arise. “Sometimes you must bargain in on your ideals to the politicians” (*F6*).

### 4.3 Category 3: Relationship built through recognition

In large organisations with mergers and cross‐professional work, the informants said that it was fundamental to recognise the profession and to talk to leaders and others.

#### 4.3.1 Strengthen the face‐to‐face recognition of subordinates

The informants said that when you lead the relationship, you involve, motivate, engage, support, and hear subordinates. Then, they feel involved in the communication and the processes that are important for the department and for them. This process is shared leadership in the form of teamwork. One leader underlined that they have skilled departments with which they share the work. “I give greater responsibility to see that it motivates, high demands on me and high demands on my departments that are directly below me” (*F6*). One informant said that she was proud of what her team had achieved today in the warning line. “Today, I can say that we are the ones who shared the tasks and knowledge together” (*F5*). The informants were concerned that they should not dominate the employees but show that their leaders listen to them. Then, they listen to the leaders differently.

#### 4.3.2 Strengthen the computerised recognition of subordinates

The informants said that the subordinates were good at documenting patients' health on the phone, but they also said that some people still panicked when they sent emails. The informants expressed that communicating via computers is a reality, but that leaders must consider that older individuals have not always learned all the programs. The leader's task is to motivate and recognise all employees. “They need to send me an email and not just come into my office because it is easier” (*N2*). All the informants said that the younger nurses can use technology in their work. “They do this, and they teach the new employees ‘quickly and precisely’” (*N3*).

### 4.4 Summary of the results

The article shows three factors influencing relationship building: in an organisation, between organisations, and between individuals. The development leaders encountered at the relation‐building (LMX) show that large organisations have both negative and positive effects on the quality of the relationships that have been and are being built. Good relationships (LMX) are a prerequisite for developing services and service provision in the health service.

## 5 DISCUSSION

### 5.1 Multidimensional relationship

In this article, we focussed on the concept relation of LMX. How do the participants talk about the relations between themselves and their co‐workers? What are the factors involved in the qualitative building of relations? In earlier articles (Frilund et al., 2022; Vasset et al., 2022), we discussed how the leadership role has changed and how the leader has influenced the processes of leading changes.

### 5.2 The relationship is built through trust and respect

The informants considered building good relationships with mutual trust and open dialogue essential for the leader. Trust was built on mutual trust. Relationships built by trust, dialogue, and recognition were affected by, among other things, organisational size, geographic location, and the number of co‐workers under the Leader's supervision. Such significant organisational changes can be complex for both leaders and employers. Leaders prioritised following up with various projects while allowing their staff to work independently. Leaders only needed to control some of the employees' actions. In recent decades, health organisations have introduced a flat structure, which has led to leaders' having an increasing number of subordinates, especially after department mergers. Therefore, leaders must rely on and trust that their subordinates do their jobs responsibly. A leader today cannot have control over everything, the informants emphasised. LMX theory describes how Leader–member relationships may develop stepwise over time (Bauer & Erdogan, 2016; Day & Miscenko, 2016; Martin et al., 2016; Vasset et al., 2012). A sequence of dyadic relations and conversational exchanges with subordinates follows this initial interaction. The leader and subordinates “test” each other to determine if they can build relational competence based on trust, respect, and high‐quality exchanges. Leading a cross‐professional team can be a challenge. Among other things, the doctors had difficulty accepting leadership from the nurses.

### 5.3 Relationship is built through dialogue

The informants started by having a monthly follow‐up discussion. They asked the employers if they had achieved the goal, what is the problem, and why the goal was not reached? What is the reason that the situation did not go as planned? The informants wanted to use discussions with subordinates to determine what went well, what went wrong and why. They defined their vision. High LMXs in communications involve physical and mental effort, emotional support, helpful and thorough feedback, and encouragement from leaders, according to Bauer and Erdogan (2016) and Peterson and Aikens (2017). Communication is essential by constantly revisiting the present situation, the participant's presence in the process, and the situation that must be addressed to implement the goal through communication and with vision. Dialogue is a two‐way communication carried out so that both parties listen to each other. The temporal perspective can lead to limited dialogue and more information from the leader (Vasset & Molnes, 2021).

### 5.4 The relationship is built through recognition

The informants said that they want shared leadership in the form of teamwork. Nevertheless, employees in health institutions still refuse to use communication technologies in which they must talk to or send emails to their leaders. Research (Bauer & Erdogan, 2016; Peterson & Aikens, 2017) has underlined that all the parties in the working relationship contribute to the development and maintenance of sociopsychological processes, such as self‐knowledge, participation, confirmation, interpersonal skills, constructive discussion, and cultural competence.

All parties in the working relationship contribute to developing and maintaining social psychological processes, such as self‐knowledge, participation, confirmation, interpersonal skills, constructive discussion, and cultural competence. The informants said that it was they who shared the tasks and knowledge. They were very concerned with not dominating the employees but with showing that they listened to them. Then, the informants said that the subordinates would listen to the informants as leaders too. One of the most important things was to listen to others and those who need to be listened to (Lervik & Vasset, 2021).

## 6 CONCLUSIONS

Long‐term leaders reflect on and share their decision‐making processes. They want to bring their subordinates along and call the process teamwork. They said that they worked to have good LMXs with their subordinates, but personnel issues remained challenging. They fought for their subordinates. Cross‐professional management and maintaining good relations with doctors were challenges. The subordinates had difficulty being led by a nurse. The informants highlighted trust, good dialogues, and confirmation as ways of maintaining good LMXs.

### 6.1 Limitations

The data were collected during the pandemic, which made face‐to‐face interviews impossible. Only eight participants were included in the study, but the results have a certain saturation level.

One limitation is that the survey group from Finland consisted only of Swedish‐speaking leaders. We might have received more nuanced results if both language groups were represented.

## AUTHOR CONTRIBUTION

Vasset has the main responsibility for the article. Frilund and Fagerstøm live in Finland and are therefore responsible for the Finnish data material. The introduction, background, and analysis were carried out by Vasset and Frilund. All authors read and approved the submitted article draft.

## CONFLICT OF INTEREST

There are none to declare.

## ETHICAL APPROVAL

The Research Ethics Committee approval number and the name of the review board who approved the study, the Norwegian Centre for Research Data (NSD). The number is 750316.

## Data Availability

The data material is a part of a larger study and will be delited.
